# Swearing, Euphemisms, and Linguistic Relativity

**DOI:** 10.1371/journal.pone.0022341

**Published:** 2011-07-20

**Authors:** Jeffrey S. Bowers, Christopher W. Pleydell-Pearce

**Affiliations:** Department of Experimental Psychology, University of Bristol, Bristol,; Macquarie University, Australia

## Abstract

Participants read aloud swear words, euphemisms of the swear words, and neutral stimuli while their autonomic activity was measured by electrodermal activity. The key finding was that autonomic responses to swear words were larger than to euphemisms and neutral stimuli. It is argued that the heightened response to swear words reflects a form of verbal conditioning in which the phonological form of the word is directly associated with an affective response. Euphemisms are effective because they replace the trigger (the offending word form) by another word form that expresses a similar idea. That is, word forms exert some control on affect and cognition in turn. We relate these findings to the linguistic relativity hypothesis, and suggest a simple mechanistic account of how language may influence thinking in this context.

## Introduction

Linguistic relativity is concerned with a profound but subtle question: Does the language you speak affect the way you think? Of course, the messages expressed in language do influence thought. That is what language is for – to implant thoughts and feelings into the minds of others. What is not so obvious, however, is whether the form of a language can also influence thought. The answer to this question is highly contentious. As Bloom and Keil put it: “The debate, as we see it, is not whether language shapes thought—it is whether language shapes thoughts in some way other than through the semantic information that it conveys. That is, the interesting debate is over whether *the structure of language* [italics theirs]—syntactic, morphological, lexical, phonological, etc.—has an effect on thought” [1].

Most of the attention – and controversy – is focused on the claim that the structure of language shapes non-linguistic thinking; so-called linguistic relativity. For instance, most languages rely on relative spatial terms to describe the relative locations of objects (e.g., the book is left/right of the pen), but in Tzeltal (a Mayan language), absolute reference terms tend to be used (e.g., uphill/downhill; the book is uphill of the pen; speakers of Tzelal live in a mountainous area). The question of interest is whether speakers of English and Tzeltal differ in their reasoning about space when language is not engaged. For evidence in support of this claim see [2]–[3]; for contrary evidence see [4]. Similar questions apply to the perception of color [5]–[6], reasoning about time [7]–[8], counting [9], memory [10], amongst other domains. For reviews and criticisms of some of this work, see [1], [11].

The present paper considers the related claim that speakers organize their thinking to meet the demands of their language during speech; so called thinking-for-speaking [12]. So for example, in English, the word friend carries no information concerning the sex of the friend, whereas in Spanish, it is inflected differently for a man (amigo) or woman (amiga). Accordingly, when talking about a friend, Spanish speakers need to contemplate their sex, whereas for English speakers, it is optional. To the extent that this morphological contrast leads speakers of the two languages to think differently while conversing, thinking-for-speaking is manifest.

Compared to linguistic relativity, the claim that languages influence thinking-for-speaking is relatively little studied, and if anything, there is a consensus that it is (trivially) true. For example, Pinker, one of the most outspoken critics of the view that language impacts on non-linguistic thinking, writes: “Whorf was surely wrong when he said that one's language determines how one conceptualizes reality in general. But he was probably correct in a much weaker sense: one's language does determine how one must conceptualize reality when one has to talk about it” [13, p. 360].

The controversy regarding this latter hypothesis is not whether people think differently while speaking, but rather, how important and interesting this observation is. Pinker stresses that the impact of thinking-for speaking is minimal, with no consequences beyond speech time. For example, comparing English and Dutch verb constructions, Pinker concludes that “it seems unlikely that the Dutch conceive of (the underlying meanings) differently from us, except at the moment that they have to express them in words” [13, p. 358]. Similarly, Levelt agrees that speaking can affect thinking: “Using a particular language requires the speaker to think of particular conceptual features” [14 p. 71]. But again, this is assumed to have minimal impact on cognition. When comparing deictic (pointing) terms across languages, he concludes: “It is highly unlikely … that English and Dutch speakers perceive distance to ego differently than Spanish and Japanese speakers. But when they prepare distance information for expression, English and Dutch speakers must represent that information in their messages in a bipartite way, whereas Spanish and Japanese speakers must use a tripartite code” [14, pp. 103–104].

By contrast, according to Slobin, thinking-for-speaking has more pervasive effects on attention, memory, and cognition generally [15]. For example, it is well established that attention plays a critical role in encoding information into episodic memory [16]. Accordingly, the fact that different languages require participants to attend to different aspects of the world when speaking may have consequences for what is experienced and remembered. Consider again the contrast between languages that describe positions using relative vs. absolute reference terms. A speaker of English may not remember whether his/her friend approached from the South, or in the direction of a distant landmark such as a mountain or the sea, as this information is not critical for the sake of conversing. By contrast, on the present hypothesis, a speaker of Tzelal would be more likely to notice and remember this aspect of their encounter. Indeed, the speaker is motivated to attend to these aspects of the world even when not speaking, as he/she must mentally encode experiences in such a way that he/she can describe them later in language, if necessary. This might be called thinking-for-potential-speaking—a case in which the distinction between linguistic relativity and thinking-for-speaking becomes blurred. Slobin describes various forms of evidence that suggest that the influence of thinking-for-speaking extends beyond the moment of speech, and shapes thought in numerous ways [15].

### Not-thinking-for-speaking

Past accounts of linguistic relativity and thinking-for-speaking tend to focus on how structural features of a language encourage specific lines of thought—e.g., attending to the sex of a friend when talking about your friend in Spanish. In the current paper we consider a situation in which structural features of a language may *discourage* specific thoughts. That is, people may avoid thinking (and conversing) about certain topics in order to avoid producing aversive word forms associated with the topic (e.g., saying aloud taboo words). On this view, it is not topic per se. that is perceived as aversive, but rather, the potential need to say aloud a given word that is. In this respect, the current hypothesis is similar to the “thinking-for-potential-speaking” hypothesis described above, but in this case, the potential speech act discourages rather than encourages certain lines of thought. We label this hypothesis “not-thinking-for-speaking”, and argue that it constitutes a version of linguistic relativity (in that thinking is affected in the absence of speaking), and that its impact is far from trivial.

Our key claim is that the phonological form of a word can directly evoke a negative emotional response, via verbal conditioning. For example, the sound of a taboo word may evoke an emotional response, independent of its semantic content. If this is correct–and much of the rest of the paper attempts to support this claim–the implications for linguistic relativity are relatively straightforward. Quite clearly we are motivated by our emotions, and we do organise our behaviour, thoughts and goals in order to avoid emotional discomfort [17]. Accordingly, to the extent that it is difficult to talk about an issue without employing emotionally conditioned words, we might be expected to avoid (not think about) the topic when possible, even when the underlying message is not negative. To the extent this is the case, it would be an example of language structure (phonology in this case) shaping thought. Indeed, such an effect would satisfy the definition of linguistic relativity outlined above [1].

In addition, this analysis suggests another (complementary) instance of word forms affecting thought; namely, the role of euphemisms in overcoming this verbal conditioning. That is, we argue that euphemisms are often useful because they allow the speaker to replace the trigger (the offending word form) by another word form that expresses the same (or similar) idea but that is not itself associated with a conditioned response. This in turn allows speakers (and listeners) to think about issues that might otherwise be avoided.

Our argument that taboo words and euphemisms are relevant to linguistic relativity claims has not previously been explored in any detail. One of the only relevant comments was made by Pinker, who dismisses euphemisms as a form of lying. He gives the example of “revenue enhancement” which has a much broader meaning than “taxes”, and argues that listeners naturally assume that if a politician had meant “taxes” he/she would have said “taxes”. As Pinker notes, “Once a euphemism is pointed out, people are not so brainwashed that they have trouble understanding the deception” [13, p. 58].

Still, there are reasons to think Pinker has been too quick to dismiss the relevance of euphemisms to the language-thought debate. For one thing, it is not true that all euphemisms are intended to mislead (lies). Some are, but many are not. The words death, urine and faeces are often replaced with passed away, number-1, and number-2 without any attempt to deceive or leave any ambiguity in the minds of the speakers or listeners. In our view, a more complete understanding of the role of euphemisms in language requires a consideration of the role of verbal conditioning.

**Figure 1:**
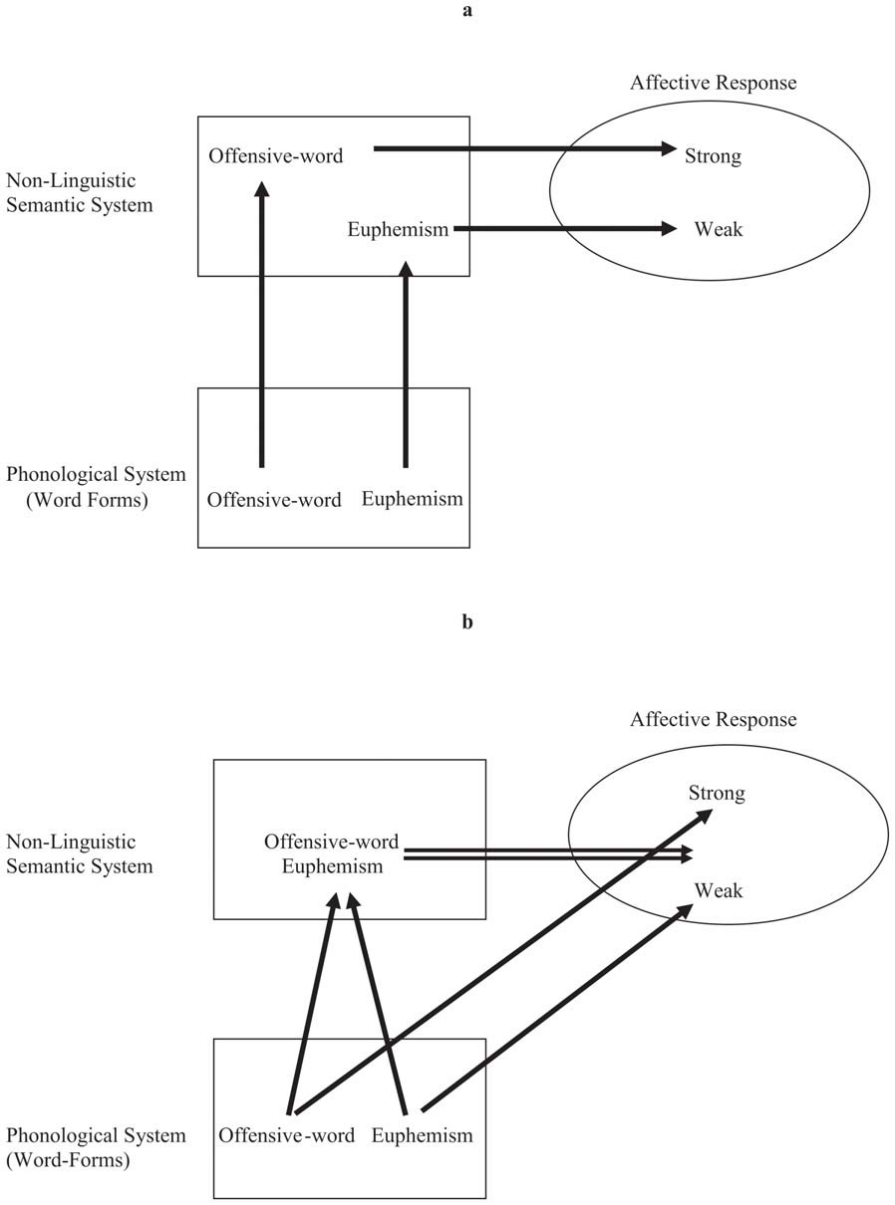
a-b. Two hypothetical ways in which swear words and euphemisms are associated with emotions. According to an approach that rejects relatively claims, the (implicit) assumption must be that word forms only influence our emotions via semantics, as in 1a. On this view, euphemisms and swear words mean different things—as indicated by their large separation in semantic space—and as a consequence, they evoke different reactions. By contrast, linguistic relativity would be supported if the structural features of a language can influence our thoughts via verbal conditioning, as in 1b. On this view, direct links develop between word forms and negative affect in response to past events in which the two stimuli co-occur. On this view, euphemisms are useful even when their meaning is very similar (or the same) to the swear word—as indicated by their small separation in semantic space—because they replace the surface form of the swear word that directly evokes negative affect.

To be more explicit about how euphemisms may develop in response to verbal conditioning, and how this is relevant to linguistic relativity claims, consider Figures 1a–b. The conventional view, according to which euphemisms are irrelevant to linguistic relativity claims, is depicted in 1a. Here, the key assumption is that word forms only influence our emotions via semantics (thoughts). That is, our emotional response to a linguistic input is only a function of the non-linguistic semantic message (mentalese) expressed by a word or passage, with the structural features of the language–such as the lexical-phonological forms of words—being irrelevant to this response (apart from the role that form plays in generating the semantic message in the first place). On this account, an offensive word and its euphemism have different emotional impact simply because they mean different things (this difference allows euphemisms to support lies, as noted by Pinker).

In Figure 1b, by contrast, word phonology can influence affect directly (unmediated by semantics) via verbal conditioning, with the semantic content of a word and its form jointly determining our emotional response. Accordingly, even if a euphemism and its offensive counterpart are close synonyms (or semantically equivalent), the two words will have different emotional impact due to the difference in their phonological forms. As a consequence, we are motivated to avoid discussing topics that involve taboo words, even when the message to be expressed is inoffensive.

Thus, one way to distinguish these two approaches is to compare emotional responses to euphemisms and their counterparts when the two words are very similar in meaning. If the items evoke very different emotional responses, this would provide support for the hypothesis that word forms are directly associated with affect.

In order to test the power of euphemisms to reduce emotional distress we focused on the two most offensive swear words in English and introduced euphemisms that were defined to have the same semantic content. The question of interest is whether they have the same emotional impact when read aloud. If euphemisms can blunt the impact of the most strongly emotionally charged words when their meaning is unambiguous, then presumably they can blunt the impact of less emotionally charged words in various semantic contexts. In the study reported here, emotional impact was measured by a physiological response, namely, electrodermal activity (EDA). There is a long history of measuring emotional impact in terms of EDA, and the validity of this measure has been supported by brain imaging studies that have examined correlations between EDA and limbic activity, in particular within the amygdala [18].

## Methods

### Ethics Statement

The study was approved by the Ethics Committee of the Psychology Department at University of Bristol. All participants provided written informed consent, and were fully debriefed at the conclusion of the experiment.

### Participants

Twenty-four volunteers took part in the study. Their mean age was 21.0 years (range 18 to 26 years). Fifteen of the participants were female.

### Design

The experiment involved two swear words printed in upper-case letters: FUCK and CUNT, and two words judged to be more neutral: GLUE and DRUM. We also constructed euphemisms for each word: the ‘F-WORD’, ‘C-WORD’, ‘G-WORD’ and ‘D-WORD’, respectively. These euphemisms were defined for the participants. Any contrast between swear words and their euphemisms will provide a measure of the efficacy of euphemisms to reduce the emotional impact of these words, whereas any contrast between the neutral words and their euphemisms will provide an assessment of whether euphemisms per se. have an impact on EDA independent of emotional responsiveness. Participants were exposed to these eight words on three occasions, organized into three blocks. The order of trials within each block was fully randomised.

Each trial began with a central fixation point presented on a computer screen for 10 s. This period served as a baseline against which effects of subsequent stimuli could be compared. The 10 s baseline was followed by one of the 8 stimuli displayed for 15 s, followed by the same fixation point used during the pre-trial phase. The post-stimulus period lasted 10 s and served to lengthen the recovery interval between adjacent trials. Participants were asked to relax during this phase. The onset of each trial was under the control of the experimenter in order to ensure that physiological measures were stable prior to the beginning of each trial.

### Physiological details

The experiment involved measurement of electrodermal activity and employed an in-house device that measured changes in skin resistance in response to an applied DC voltage source. The device was set to DC, and the output signal was not subject to any RC filtering. Outputs from the device were passed to the analogue inputs of a Neuroscan amplifier. The amplifier had a high pass setting of DC, and a low pass filter of 30 Hz. Data were acquired at 200 Hz with a gain of 250 (22 mv full-scale resolution). EDA recording employed non-polarising Ag/AgCl electrodes located on the volar surface of the first phalanx of the first and third fingers of the left hand.

### Procedure

Participants were warned that they would be exposed to swear words, and an opportunity to withdraw from the study was given. No volunteer selected this option and this likely reflects the fact that the adverts for the experiment contained a warning about the general nature of the study. Participants were informed about the words, euphemisms, and neutral words, and instructed to read aloud each item once as soon as it was presented on the computer screen. After reading aloud the word they were instructed to respond “YES” if the referent was a swear word, and “NO” otherwise in order to insure that the participants understood that the C-Word and F-Word referred to swear words, and the G-WORD and D-WORD did not. Vocal responses were monitored by the experimenter located in an adjacent room. The entire procedure lasted around 1 hour and the recording phase lasted about 20 minutes.

## Results

**Figure 2:**
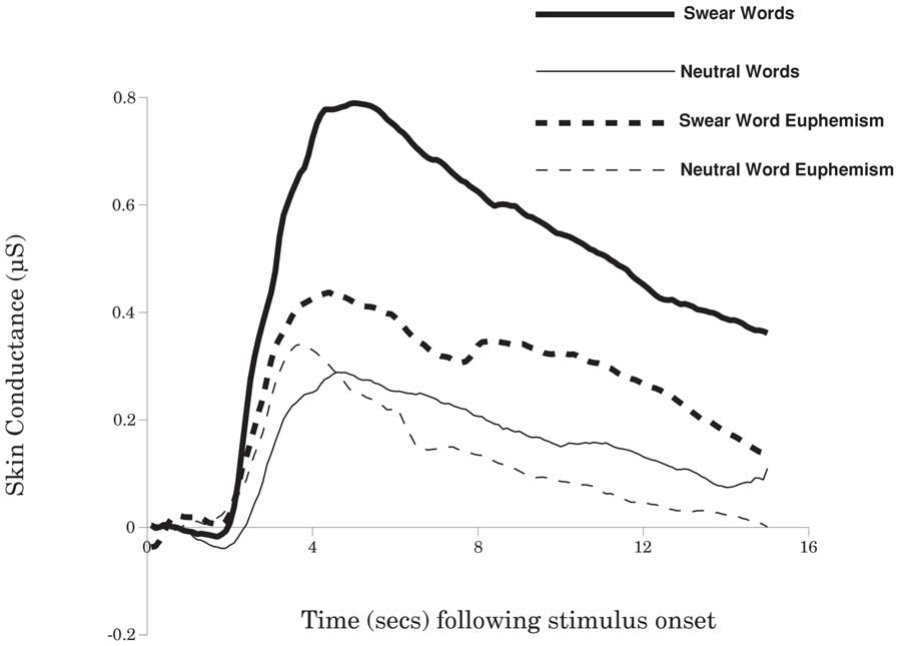
Mean electrodermal activity (EDA) invoked by stimulus onset across the four conditions.

Participants made no naming or categorization errors during the experiment. Figure 2 displays mean EDA across conditions. The data indicate that real swear words invoked the greatest electrodermal response, followed by euphemistic versions of the swear words. Neutral words and their euphemism equivalents produced a much smaller overall response. Data were analysed in two ways. First, a mean response for each of the four conditions (averaged across trials) was derived over a period from 3 s to 6 s following stimulus onset. The first 3 s period was avoided due to presence of participant's initial vocal response. Each mean amplitude was itself baselined with respect to a 1 s period prior to stimulus onset (word or euphemism). Henceforth we term this measure 'trial amplitude' (TA). However, EDA values are known to vary considerably across participants [19]. Thus, a second measure involved normalising EDA measures by conversion to standard (z) scores. The normalisation was applied after condition averages were truncated to a 10 Hz sampling rate. Each of the four condition averages (within participant) supplied 30 data points (3 s×10 Hz  = 30) and z scores were based upon calculation of standard deviations across the entire range of 120 points within the measurement window (30 points×4 conditions). Thus while the overall mean of the four conditions (within participant) was equal to zero, between condition differences were preserved. After this correction was applied, a normalised trial amplitude (NTA) was derived in the same manner as described earlier for TA.

Data analyses employed a one-way analysis of variance with four levels: swear words, swear word euphemisms, neutral words, and neutral word euphemisms. For the TA measure, the main effect of condition was significant: F(3,69) = 5.67, P = 0.019 (Greenhouse-Geisser corrected). Critically, a planned contrast between swear words and their euphemism controls was significant, with larger TA responses to the swear words, t = 2.25, P<0.05, whereas the contrast between neutral words and their neutral euphemism controls was not t<1. Analysis involving the NTA variable yielded a similar pattern of results, with a significant main effect of conditions, F(3,69) = 10.69, P<0.001 (Greenhouse-Geisser corrected). Post hoc contrasts between means followed the exact same pattern as described for the TA variable.

## Discussion

The results of the study are clear-cut and perhaps unsurprising; people find it more stressful to say aloud a swear word than its corresponding euphemism. Presumably this is why euphemisms are employed in so many contexts and in all languages (In Swedish the word jävlar [devils] is considered quite harsh and tends to be substituted by the form similar word järnvägar [railroads]. Railroads!). What is surprising, however, is that little or no consideration has been given to this phenomenon and its relevance to the long-standing debate concerning the relation between language and thought.

On the present hypothesis, the emotional reactions observed in the laboratory reflected, in part, a conditioned response to the sounds of the swear words. This learning might also occur between the visual form of swear words and affect, with little generalisation – e.g., our reactions are much reduced to the orthographically related letter string fcuk (a brand name for clothing marketed in the UK). To the extent that it is difficult to talk about an issue without employing emotionally conditioned words, we might be expected to avoid (not think about) the topic when possible. Euphemisms are, in this view, effective because they replace the trigger (the offending word form) by another word form that expresses the same (or similar) idea, allowing the relevant message to be communicated without triggering the emotional response. This in turn allows speakers (and listeners) to think about issues that might otherwise be avoided; linguistic relativity par excellence.

Although this hypothesis is novel, the claim that swear words are represented differently than most other words, with direct links between their form and emotional systems, is familiar in neuropsychological studies of language. For instance, swearing is frequently one of the few language skills preserved in severely aphasic patients, and it is prevalent in the disorder Gilles de la Tourette in which 25–50% of the patients swear involuntarily [20]. Based on brain stimulation studies in humans [21] and behavioural studies following surgery [22], Robertson, Dornan and Trimble [23] proposed that the cingulate cortex – a critical component of the limbic system involved in coding for emotions – plays an important role in mediating emotionally charged language. Indeed, based on earlier observations on animal vocalizations, Robinson [24] proposed that two brain systems are involved in language: an older system that terminates in the cingulate gyrus capable of emotive speech (a system shared by humans and non-humans), and a newer cortical system involved in mediating complex (generative) language. It is the preservation of the older sub-cortical system in aphasia that would account for the preservation of swearing in some aphasic patients, and hyperactivity of the older system that may account for the involuntary swearing in Tourette's syndrome (for detailed review of the neuropsychology of swearing, see [20]). For the present purpose, however, the important point is that these findings are consistent with the claim that the forms of swear words have direct access to emotional centres of the brain, unmediated by higher-level cognitive analysis. This conclusion is supported by various behavioural studies that also suggest that stimuli can evoke affective responses independently of cognitive (semantic) analysis [e.g., 25–27, but see 28].

And why should this surprise anyone? The suggestion that the sounds (and spellings) of words can be associated with emotional responses in humans seems little different from lights, puffs of air, bells or whistles acting as conditioned stimuli that evoke conditioned responses in a rat. Indeed, the phonology and orthography of words are not only directly connected with semantics, but also with each other, with syntax, and they may even have direct associations with motor systems [29]. Accordingly, there seems no a priori reason to assume that word forms must access affect via semantics. Interestingly, the claim that verbal conditioning plays a role in constraining thinking has been advanced in another context as well (thinking about motion; [30]).

Still, a critic of our hypothesis might claim just this, and argue that the contrasting emotional responses to the swear words and their euphemisms reflect contrasting semantic and/or pragmatic distinctions rather than form distinctions. For example, swear words tend to be spoken with the intention to evoke a response in the listener, whereas euphemisms are spoken with the intention to communicate the same idea while minimising the emotional response. Accordingly, our muted response to the f-word, for example, could reflect an understanding that the speaker intends not to offend – a conceptual rather than form difference. On this view, euphemisms are effective not because they are lies (although some can be characterised this way), but because the speaker revealed his/her desire not to offend by speaking in euphemisms, and the listener, rather then being deceived, is highly sensitive to the speakers intentions (for similar interpretations, see [31]–[34]; for a related interpretation based on a strategic use of indirect language, see [35]). This would not constitute an instance of language constraining thought, but rather, the fact that different words mean different things.

Indeed, it is almost certainly the case that offensive words and their euphemistic mates are often interpreted differently, and that these differences play a role in modulating our affective responses. But this analysis does not undermine our hypothesis. In order to conclude that the euphemisms are irrelevant to linguistic relatively claims, it must be argued that conceptual contrasts are entirely responsible for the present findings, with verbal conditioning playing no role. More generally, in order to reject linguistic relativity claims, it must be assumed that the emotional impact of language is the product of our interpretations of utterances, with no form influences.

Although we cannot rule out the claim that semantic/pragmatic effects are entirely responsible for our findings, a number of related considerations pose a serious challenge for this view. Firstly, in the present study, the swear words and the euphemisms were defined to be equivalent, just as DRUM and the D-WORD were defined to be equivalent. It is difficult to argue that the semantics of DRUM and the D-WORD are different when they are defined to be the same, and this would seem to apply to the swear words and their euphemisms as well. Consider what it would mean to argue that the contrast between the F-WORD and its counterpart is semantic. It would imply that the full meaning of the swear word can only be accessed by its complete word form, and that it is not possible to access its meaning (and associated affect) by introducing a synonym. That is, it would have to be argued that the semantics of swear words cannot be separated from their form. In which case, there is no point in asking whether the phonology of words can impact on thinking in some way other than through the semantic information that it conveys [1].

The more powerful challenge to our hypothesis, however, is that the contrasting responses only reflect pragmatic distinctions. No one yells “F-WORD!” when angry. Again, we do agree that these pragmatic contrasts play a role in modulating our emotional responses to words in many circumstances, but in our experiment, participants were instructed to say the swear words and euphemisms aloud in the context of a psycholinguistic experiment. In this situation, it is difficult to argue that the contrasting results reflected differences in the intent of the speakers (or the participants' concern regarding the experimenter's response). In a similar way, when developing and discussing this project, we both found it more comfortable to use the c-word between ourselves, even though swear words would have been used in the context of developing a psycholinguistic study on euphemisms. It seems unlikely that our preference for the c-word reflects a concern that we would otherwise offend. We also prefer to write the c-word (and expect you prefer to read the c-word), despite the fact that the word is being used in an academic context, and despite the fact that you are quite likely reading these words to yourself. These observations do not sit well with an account in which pragmatic/semantic contrasts are entirely responsible for the present findings.

A nice demonstration that our reactions to swear words are not entirely the product of pragmatics is that we react to accidental productions of swear words. For example, on December 6, 2010, the BBC broadcaster James Naughtie said the following on Radio 4 in the UK: “First up after the news we are going to be talking to Jeremy Cunt ...er Jeremy Hunt! - the Culture Secretary about the art of Broadband”. Shortly afterwards, Andrew Marr hosted a discussion about the Freudian slip, and proceeded to make the same mistake. See: http://www.guardian.co.uk/media/2010/dec/06/james-naughtie-today-jeremy-hunt?INTCMP=SRCH. A striking feature of these (obviously unintentional) mistakes was that offense was taken by the listening audience, and the BBC felt the need to apologize. Indeed, neither programme was available on the BBC's iPlayer (a website that would normally allow listeners to listen to these shows again). In a statement on its complaints website, the BBC wrote: “James and Andrew regret what happened and have both apologised for their verbal tangles on air. These instances both involved a slip of the tongue during a live broadcast, and we apologise for any offence caused.” James Naughtie later commented on Radio 4 “We know from emails that some of you thought it was funny, and others were very offended… I'm very sorry to those of you who thought it wasn't what you wanted to hear over your breakfast. Neither did I.” Of course, if our emotional reactions were entirely due to the pragmatics of the situation, then no offense would have been taken, and no apologies offered.

A number of cross-linguistic studies also challenge the claim that the emotional impact of swear words is entirely the product of semantics and/or pragmatics**.** For example, Bond and Lai [36] found that bilingual speakers feel more free discussing embarrassing topics in their second language in a laboratory setting. An illustration of this was earlier reported by Kwok and Chan [37]; they reported the case of a Chinese student who would not confess to a priest in his native Cantonese because “it would hurt too much” [p. 70]. Instead, he confessed in his second language, English. In both of these situations, a similar message was communicated more easily in a second compared to native language, consistent with the claim that affect is linked with language per se.

Perhaps more relevant, a number of studies have found that taboo words often generate more anxiety in participants when spoken (or written) in their first language [38]–[42]. Most strikingly, Harris and colleagues [43] found increased EDA responses to taboo words presented in the participants' first (Turkish) compared to second (English) language. The claim that the contrasting EDAs are the product of semantic analyses amounts to the claim that bilingual Turkish speakers understand translation equivalent terms, such as the English phrase “oral sex” and the Turkish word “masturubasyon” (an example taken from their paper) differently. This despite the fact that the speakers were familiar with the words in both languages, and despite the fact that Harris and colleagues [43] reported no effect of word familiarity on EDA responses. Similarly, to attribute the different EDA responses to pragmatic contrasts seems unlikely given that the swear words in both languages were understood to be taboo words (unlike euphemisms). Although the above authors did not relate their cross-linguistic findings to issues of linguistic relativity, the results clearly parallel our own, and in our view, lend support to the claim that the impact of swear words is due, in part, to the phonological (or orthographic) forms of these words evoking negative emotional states (independently of semantic analysis).

In sum, it seems clear that we can introduce synonyms that are functionally equivalent to familiar words: we can coin the term “blap” to refer to a “pencil”, D-WORD for drum, or stipulate that X stands for Y. However, as the current findings demonstrate, we cannot define a word that functions like “fuck” (even if it is called the F-WORD, or is a translation equivalent in a second language). It is not enough to tell speakers that they mean the same thing – the words need to have the same sound in order to evoke the same response. And this is the point – the phonological forms of words do matter. This is not to deny that conceptual factors play a key role in modulating our emotional responses to these words, but according to the present hypothesis, the emotional force of words cannot be reduced to these conceptual distinctions.

But is this relevant to linguistic relativity claims? It is if we accept the definition of linguistic relativity that we started with: “The debate, as we see it, is not whether language shapes thought—it is whether language shapes thoughts in some way other than through the semantic information that it conveys. That is, the interesting debate is over whether *the structure of language* [italics theirs]—syntactic, morphological, lexical, *phonological* [italics ours], etc.—has an effect on thought” [1]. The only missing link in our argument is the claim that thoughts and motivations are affected by emotions – which seems transparently true. Indeed, this link helps explain why Bond and Lai [36] found that bilingual speakers were more free discussing embarrassing topics in their second compared to their first language. It is not the content of the messages differed in the two languages; it is the phonological forms of the words that differed.

An alternative criticism of the present hypothesis might also be advanced. That is, the present findings might be taken as evidence in support of linguistic relativity, but only in a trivial sense. As noted above, thinking-for speaking is sometimes considered a trivial example of language impacting on thought because it is only assumed to impact on thinking during the speech act, with no lingering effects [13]–[14]. There are reasons to doubt this conclusion [14], [44], but in any case, a critic of the present hypothesis might be tempted to make a similar claim for the present case. However, we are claiming that the word forms discourage conversations and associated thoughts from occurring in the first place. The consequence of avoiding a thought or conversation is hard to quantify, but presumably, in some circumstances, the costs are long lasting and profound. In no sense can the impact be described as transient.

Of course, the fact that euphemisms are readily available in all languages makes it easier for speakers to express unpleasant thoughts while avoiding offensive words (on the current argument, this is part of the reason euphemisms are coined in the first place). But the relevant issue is not whether verbal conditioning prevents thinking about unpleasant topics, but rather, whether thoughts can be influenced or biased by the forms of words. We think we have made a *prima fascia* case that the phonological forms of words do indeed impact on thinking, as have others [36] – although the relevance of previous findings to these questions was not appreciated.

To conclude, let us offer an illustration of the possible import of word forms in affecting thought and action. The following conversation was described by Pilger [45]:

At the Paris arms fair, I asked a salesman to describe the working of a “cluster grenade” the size of a grapefruit. Bending over a glass case, as one does when inspecting something precious, he said, “This is *wonderful*. It is state of the art, unique. What it does is discharge copper dust, very very fine dust, so that the particles saturate the objective…”.

“What objective?” I asked.

He looked incredulous. “Whatever it may be”, he replied.

“People?”.

“Well, er…. If you like.”

The only pleasure to be had at these events is in helping the salesmen relieve their verbal constipation. They have the greatest difficulty saying words like “people” and “kill” and “maim”. (p. 101)

It is doubtful there is confusion in the minds of buyers or sellers about the function of weapons. Nevertheless, on our account, the euphemisms allowed business to be conducted with minimal discomfort.

## Summary

In sum, we would like to advance the hypothesis that the strong EDA responses to swear reflect, in part, form-affect associations. As noted above, the claim that word forms can directly evoke an emotional responses is consistent with various neuropsychological [20] and cognitive [27] evidence that verbal (and nonverbal) stimuli can be closely associated with emotional systems, perhaps independently of semantic systems. In our view, euphemisms are effective because they replace the trigger (the offending word form) by another word that is similar conceptually. This, in turn, might allow us to discuss the same issues without the offending words, making conversation, associated thoughts, and related behaviour more likely than otherwise would be the case. Such an outcome satisfies the definition of linguistic relatively: Word forms, in and of themselves, exerting some control on affect and cognition in turn.

Of course, this is only a first attempt at characterizing the mental processes that support our differing responses to swear words and euphemisms; further work is required before any strong conclusions are warranted. But this caveat does not undermine what we see as the main contribution of this paper, which is to highlight the potential relevance of euphemisms and verbal conditioning to these longstanding questions.
